# UGT1A and TYMS genetic variants predict toxicity and response of colorectal cancer patients treated with first-line irinotecan and fluorouracil combination therapy

**DOI:** 10.1038/sj.bjc.6605776

**Published:** 2010-07-13

**Authors:** E Martinez-Balibrea, A Abad, A Martínez-Cardús, A Ginés, M Valladares, M Navarro, E Aranda, E Marcuello, M Benavides, B Massutí, A Carrato, L Layos, J L Manzano, V Moreno

**Affiliations:** 1 Medical Oncology Service, Institut Català d'Oncologia, Hospital Germans Trias I Pujol. C/Ctra. Canyet s/n, Badalona 08916, Spain; 2 Fundació Institut d'Investigació en Ciències de la Salut Germans Trias i Pujol (FIGTP). C/Ctra. De Can Ruti, camí de les escoles s/n, Badalona 08916, Spain; 3 Medical Oncology Service, Complejo Hospitalario Universitario Juan Canalejo, C/Lugar Xubias De Arriba 84, A Coruña 15006, Spain; 4 Medical Oncology Service, Institut Català d'Oncologia, Hospital Duran I Reynals, Avda. Gran Vía s/n Km 2.7, L'Hospitalet de Llobregat 08907, Spain; 5 Medical Oncology Service, Hospital Universitario Reina Sofía, C/Menéndez Pidal s/n, Córdoba 14004, Spain; 6 Medical Oncology Service, Hospital de la Santa Creu I Sant Pau, C/Sant Antoni Maria Claret, 167, Barcelona 08025, Spain; 7 Medical Oncology Service, Hospital Carlos Haya, Avda. Carlos Haya s/n, Málaga 29010, Spain; 8 Medical Oncology Service, Hospital General Universitario de Alicante, Avda. Pintor Baeza s/n, Alicante 03010, Spain; 9 Medical Oncology Service, Hospital General Universitario de Elche, Camí Almassera 11, Elche 03203, Spain; 10 Biostatistics and Bioinformatics Unit, Institut Català d'Oncologia, Hospital Duran i Reynals, Avda. Gran Vía s/n Km 2.7, L'Hospitalet de Llobregat 08907, Spain; 11 Institut d'Investigació de Bellvitge, Feixa Llarga s/n, L'Hospitalet de Llobregat 08907, Spain; 12 Department of Clinical Sciences, University of Barcelona, Feixa Llarga s/n, L'Hospitalet de Llobregat 08907, Spain

**Keywords:** colorectal, 5FU, irinotecan, toxicity, polymorphisms

## Abstract

**Background:**

The impact of thymidylate synthase (TYMS) and UDP-glucoronosyltransferase 1A (UGT1A) germline polymorphisms on the outcome of colorectal cancer (CRC) patients treated with irinotecan plus 5-fluorouracil (irinotecan/5FU) is still controversial. Our objective was to define a genetic-based algorithm to select patients to be treated with irinotecan/5FU.

**Methods:**

Genotyping of TYMS (5′TRP and 3′UTR), UGT1A1^*^28, UGT1A9^*^22 and UGT1A7^*^3 was performed in 149 metastatic CRC patients treated with irinotecan/5FU as first-line chemotherapy enrolled in a randomised phase 3 study. Their association with response, toxicity and survival was investigated by univariate and multivariate statistical analysis.

**Results:**

TYMS 3TRP/3TRP genotype was the only independent predictor of tumour response (OR=5.87, 95% confidence interval (CI)=1.68–20.45; *P*=0.005). UGT1A1^*^28/^*^28 was predictive for haematologic toxicity (OR=6.27, 95% CI=1.09–36.12; *P*=0.04), specifically for neutropenia alone (OR=6.40, 95% CI=1.11–37.03; *P*=0.038) or together with diarrhoea (OR=18.87, 95% CI=2.14–166.67; *P*=0.008). UGT1A9^*^1/^*^1 was associated with non-haematologic toxicity (OR=2.70, 95% CI=1.07–6.82; *P*=0.035). Haplotype VII (all non-favourable alleles) was associated with non-haematologic toxicity (OR=2.11, 95% CI=1.12–3.98; *P*=0.02).

**Conclusion:**

TYMS and UGT1A polymorphisms influence on tumour response and toxicities derived from irinotecan/5FU treatment in CRC patients. A genetic-based algorithm to optimise treatment individualisation is proposed.

Despite the development of novel anticancer agents, such as bevacizumab, cetuximab or panitumumab, the backbone of metastatic colorectal cancer (CRC) treatment is based on the combination of 5-fluorouracil (5FU) (±leucovorin), oxaliplatin and irinotecan (O'Neil and Goldberg, 2008). For first-line treatment, oxaliplatin+5FU or irinotecan+5FU are indistinctly used. With regard to irinotecan-based schedules, about 50% of patients respond to therapy but half of them are expected to experience some kind of severe toxicity, mainly diarrhoea and neutropenia, resulting either in dose reduction, treatment withdrawal or dying (Slichenmyer *et al*, 1994; Douillard *et al*, 2000). The significant patient variability in response and toxicity and the existence of common genetic variants within genes important to their metabolism and/or activity, highlights the relevance of pharmacogenetic studies that could help to identify individuals with a higher probability of developing severe toxicity or an increased response rate. The efficacy of irinotecan depends on activation by carboxyesterases to form the active metabolite SN38 (Kawato *et al*, 1991; Sanghani *et al*, 2003). To facilitate its elimination, SN38 is glucoronidated by UDP-glucoronosyltransferase 1A (UGT1A) enzymes, mainly by the hepatic UGT1A1 but also by UGT1A7 and UGT1A9 (Ciotti *et al*, 1999; Gagne *et al*, 2002). Genetic variation in UGT1A1 enzyme was first related to the Gilbert's syndrome, in which the homozygous variant genotype UGT1A1^*^28/^*^28 is responsible for a less efficient bilirrubin glucoronidation (Monaghan *et al*, 1996). Later, a series of publications demonstrated that genotyping of UGT1A1^*^28 could also serve to predict irinotecan-associated severe toxicity. Thus, in 2005, the US Food and Drug Administration suggested that patients homozygous for UGT1A1^*^28 should receive a reduced starting dose of irinotecan by at least one level. However, given that the UGT1A1^*^28/^*^28 genotype does not explain many cases of severe toxicity, other factors, genetic and nongenetic, should be analysed. Results from several studies have shown that other genetic variants within the UGT1A1 sequence and also in UGT1A7 and UGT1A9 isoforms have to be taken into account (Carlini *et al*, 2005; Han *et al*, 2006; Cecchin *et al*, 2009). Moreover, Thymidylate synthase (TYMS) polymorphisms located in 5′UTR region are, probably, of the most broadly studied genetic variants in the field of pharmacogenetics in CRC (Marsh, 2005). It is quite accepted that the 3TRP allele is associated with increased levels of the chemotherapeutic target TYMS (mRNA, protein or activity depending on the different studies) (Kawakami *et al*, 2001; Etienne *et al*, 2002; Ishida *et al*, 2002) and this fact has been associated with poorer outcome in patients treated with 5FU-based regimens both in the adjuvant and in the metastatic setting (Iacopetta *et al*, 2001; Pullarkat *et al*, 2001). Also, the 6 bp deletion in the 3′UTR of *TYMS* gene has been correlated with increased message instability and consequently, lower levels of TYMS mRNA (Mandola *et al*, 2004).

The aim of this study was to elucidate which UGT1A polymorphisms or combination of them should be analysed to better predict severe toxicity in colorectal patients treated with irinotecan-based first-line chemotherapy. We also studied the influence of their haplotypes and of TYMS variants.

## Materials and methods

### Study design and patients

A representative sample (*N*=149, 43%) of patients from a multicentre, prospective, randomised phase III study (*N*=346) in first-line CRC treatment, carried out by the Spanish Cooperative Group for the Treatment of Digestive Tumours (TTD Group), was included in this analysis. Patients were selected based on availability of blood samples. The study compared weekly irinotecan (80 mg m^−2^) plus high-dose 5FU (FUIRI) *vs* biweekly irinotecan (180 mg m^−2^) plus 5FU/LV (FOLFIRI) as first-line chemotherapy for patients with metastatic CRC. The conclusions of the trial and details on eligibility criteria, treatment schedules, guidelines for dose-modifications and all the study details have already been published. In summary, FUIRI represented a less complex and equivalent alternative to the standard FOLFIRI regimen (Aranda *et al*, 2009). Median follow-up of the study was 17.2 months (95% confidence interval (CI)=15.7–18.5). Toxicities were grouped as haematologic (neutropenia, leucopenia, thrombocytopenia and anaemia) or non-haematologic (diarrhoea, nausea, vomiting, asthenia, anorexia, mucositis and non-neutropenic infection). Grade 3–4 toxicity was considered as severe. The pharmacogenetic study protocol was approved by the local ethics committee, and all subjects gave informed consent before participating in the study.

### Genetic analyses

**Figure 1:**
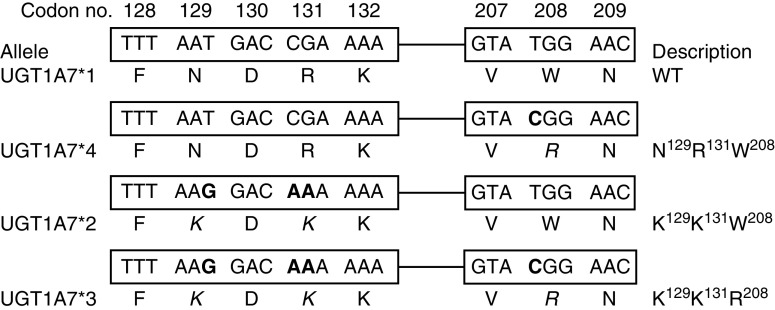
Graphic representation of three of the polymorphic sites (codons 129, 131 and 208) studied on the human UGT1A7 exon 1 sequence. The corresponding alleles are shown. Changes in base pairs are shown in bold, whereas changes in amino-acid sequence are shown in italics.

Genotypes were determined in DNA extracted from peripheral blood samples using the QiAmp DNA Blood Mini kit (Qiagen, Valencia, CA, USA) according to manufacturer's instructions. The TS promoter region polymorphism (5′VNTR) was analysed through standard PCR method and the 3′UTR polymorphism was analysed by GeneScan, both as previously described (Martinez-Balibrea *et al*, 2008). UGT1A1^*^28 and UGT1A9^*^22 were analysed by automatic sequencing using forward (5′-CTGAAAGTGAACTCCCTGCTACCT-3′ and 5′-GCTTCTAAACTTAACATTGCAGCACA-3′, respectively) and reverse (5′-GCAGGAGCAAAGGCGCCATG-3′ and 5′-CTGGGAATCTAAGCTCCTATGATACA-3′, respectively) nested primers with an ABI Prism 3100 DNA Analyzer (Applied Biosystems, Foster City, CA, USA). To define UGT1A7^*^1, UGT1A7^*^2, UGT1A7^*^3 and UGT1A7^*^4 alleles, we performed an allelic discrimination assay in an ABI Prism 7000 system (Applied Biosystems) for UGT1A7 N^129^K R^131^K and W^208^R variants (Guillemette *et al*, 2000). A graphic representation of these polymorphic sites can be found in Figure 1. As a control, we also performed an allelic discrimination assay for UGT1A7-57 (T → G), which is completely linked to UGT1A7 W^208^R (Lankisch *et al*, 2005). We used primers and probes already reported (Lankisch *et al*, 2005). All primers and probes were designed and/or validated by using the Primer Express software v2.0 from Applied Biosystems.

### Statistics

In this retrospective analysis within a prospective trial, we evaluated the association between UGT1A genotypes/haplotypes and TYMS genotypes and toxicity (severe toxicity at first cycle and at the end of treatment), response rate, time to progression (TTP) and overall survival (OS). Each polymorphism was tested to ensure that it fitted Hardy–Weinberg equilibrium. The associations between categorical variables were assessed by *χ*^2^-tests or Fisher's exact test whenever required. Also, logistic regression models were used to estimate the associations of genotypes and toxicity or response to treatment. Each polymorphism was analysed as a three-group categorical variable (codominant model), and also other restricted inheritance models were used (log-additive, dominant and recessive). Linkage disequilibrium (LD) between polymorphisms was calculated and the analysis of inferred haplotypes was also performed. The web tool SNPstats (Sole *et al*, 2006) was used for the genetic analyses and some analyses were also performed in R (http://www.r-project.org). The Kaplan–Meyer plots were used to estimate TTP and OS curves. The association of genetic variants and clinical parameters with TTP and OS was assessed with the log-rank test, and also hazard ratios (HR) and their 95% CIs were estimated from univariate and multivariate Cox proportional hazards regression models. In general, differences were considered statistically significant when two-sided *P*-values were <0.05. However, to account for multiple comparisons when analysing polymorphisms or haplotypes, a Bonferroni correction was applied and significant differences were considered for *P*-values <0.005. This assumes 10 independent comparisons and should suffice for a nominal 0.05 type I error protection for five independent SNPs that were analysed in several correlated inheritance models and haplotypes.

## Results

### Allelic, genotypic and haplotypic frequencies and LD analysis

**Figure 2:**
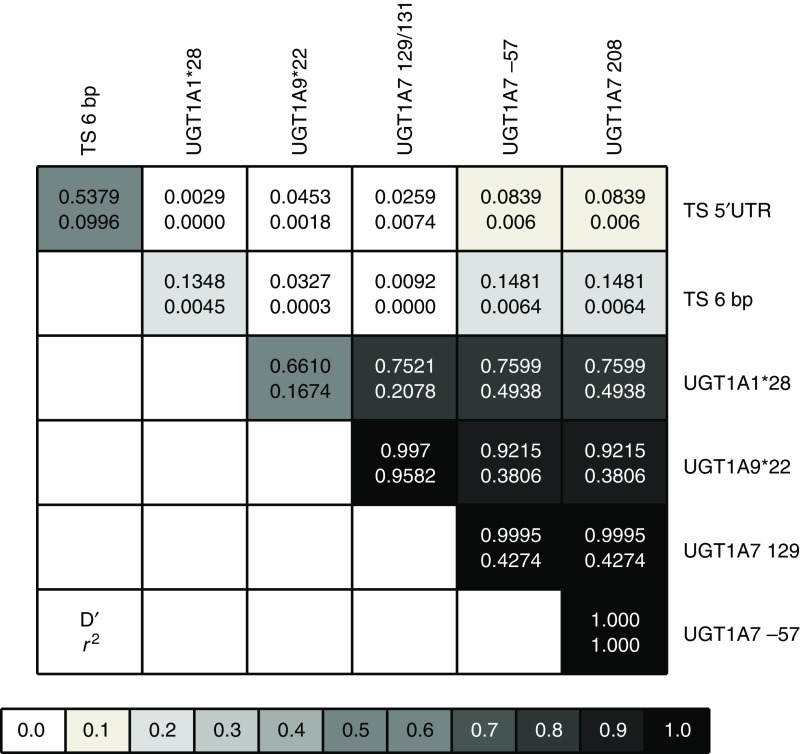
Pairwise linkage disequilibrium relationships between TYMS and UGT1A variants. The Lewontin's coefficient *D*′ and the correlation coefficient *r*^2^ are reported. A grey scale represents statistical significance of the association.

Allelic and genotypic frequencies were calculated for all genetic variants and are shown in Table 1. All variants were in Hardy–Weinberg equilibrium. As previously reported (Lurje *et al*, 2008), TS 5′TRP and TS 3′UTR polymorphism were in LD (*D*′=0.538; *r*^2^=0.1) but no linkage was observed between these and UGT1A variants (Figure 2). UGT1A7 W^208^R and UGT1A7-57 variants were totally linked as described before (Lankisch *et al*, 2005) (*D*′=1.00; *r*^2^=1.00) and a high LD was observed between them and UGT1A7 N^129^K variant (*D*′=0.99; *r*^2^=0.427). UGT1A9 and UGT1A7 variants were more linked to each other (0.922<*D*′<1.00; 0.381<*r*^2^<0.958) than to UGT1A1^*^28 (0.661<*D*′<0.76; 0.167<*r*^2^<0.494) (Figure 2). UGT1A haplotypes I, II, III and VII were in a frequency >5% and therefore they were considered in response, toxicity and survival analysis. Haplotype VII harbours all UGT1A variants previously associated with severe toxicity (UGT1A1^*^28, UGT1A9^*^1 and UGT1A7^*^3) (Table 1).

### Response to therapy

A total of 147 patients were evaluable for response. The majority of them (47.7%) attained a partial response or tumour stabilisation (34.9%), whereas 5.4% had a tumour progression and 10.7% a complete response. These response rates were similar to those obtained in the whole study (Aranda *et al*, 2009). Patients were grouped as responders (R, 58.4%) if they had a partial or a complete response, or as no responders (NR, 41.6%) if they had a tumour stabilisation or progression. Seventy four percent of patients received more than three cycles of chemotherapy. Among all clinical variables, poor tumour differentiation and number of chemotherapy cycles received ⩽3 were negatively associated with tumour response (*P*=0.023 and 0.038, respectively). Patients whose primary tumour was located in the rectum were more likely to respond to chemotherapy (64.1 *vs* 49.1% for colon tumours), although these differences were not statistically significant (*P*=0.076). All results for the association of clinical variables and response to therapy are shown in Table 2. When genetic variants were taken into account, only TS 5′TRP polymorphism was strongly associated with response to therapy. As shown in Table 3, 79% of patients harbouring the 2TRP/2TRP genotype responded to therapy, whereas 61 and 40% of patients heterozygous and homozygous for the 3TRP allele, respectively, did so (*P*=0.0024). None of the UGT1A variants or haplotypes was associated with response to therapy. In the multivariate analysis, TS 5′TRP genotype, grade of tumour differentiation and number of chemotherapy cycles were independent predictive factors of response (Table 4).

### Toxicity

The most frequent severe toxicities were as follows: diarrhoea (30.2%), neutropenia (20.8%), vomiting (5.37%), mucositis (4.03%), nausea (2.68%), leucopenia (2.01%), febrile neutropenia (1.34%) and thrombocytopenia (0.67%). Patients who received FOLFIRI had greater rates of severe neutropenia (30 *vs* 11%), whereas patients who received FUIRI had greater rates of severe diarrhoea (40 *vs* 18%). Although in this cohort there were no treatment-related deaths (there were 6 treatment-related deaths in the global study), 10 patients (6.7%) experienced severe neutropenia and diarrhoea at the same time. To make the genetic analysis more comprehensive, we analysed those genotypes previously associated with severe toxicities (i.e., UGT1A1^*^28/^*^28, UGT1A9^*^1/^*^1 and UGT1A7^*^3/^*^3) *vs* the rest of the genotypes for a given polymorphism (e.g., UGT1A1^*^28/^*^28 *vs* UGT1A1^*^28/^*^1 and UGT1A1^*^1/^*^1). The detailed analysis can be found in the Supplementary Table S1 (online only). Given that the occurrence of diarrhoea and neutropenia at the same time is considered as a life-threatening irinotecan-related toxicity, we also studied its relationship with all the UGT1A variants. At the end of the first cycle of treatment, patients harbouring either the UGT1A1^*^28/^*^28, all or some non-favourable UGT1A genotypes (either UGT1A1^*^28/^*^28 or UGT1A7^*^3/^*^3 or UGT1A9^*^1/^*^1), or the haplotype VII were at a higher risk of developing non-haematologic toxicity. Patients with UGT1A1^*^28/^*^28 or some unfavourable UGT1A genotype had higher rates of severe diarrhoea (Table 5). At the end of treatment, UGT1A1^*^28/^*^28 and all non-favourable genotypes were associated with haematologic and non-haematologic toxicities, severe neutropenia and diarrhoea and both. Patients with the UGT1A9^*^1/^*^1 genotype were more likely to develop severe non-haematologic toxicity and diarrhoea, whereas UGT1A7^*^3/^*^3 genotype was also associated with the occurrence of neutropenia and diarrhoea simultaneously. Patients harbouring some of the unfavourable genotypes or the haplotype VII were at a higher risk of developing non haematologic toxicity and severe diarrhoea (Table 5). In a multivariate logistic regression analysis, UGT1A1^*^28/^*^28 genotype was an independent predictive factor of haematologic toxicity and severe neutropenia with or without diarrhoea at the end of treatment. At the end of the first cycle of chemotherapy, this genotype predicted for severe diarrhoea and non-haematologic toxicity (Table 4). UGT1A9^*^1/^*^1 genotype and age older than 65 years were independent predictive factors of non-haematologic toxicity at the end of treatment. UGT1A9^*^1/^*^1 and UGT1A7^*^3/^*^3 were associated with higher risk of severe diarrhoea at the end of the first cycle of chemotherapy (Table 4). It is worth highlighting that those patients with TS 2TRP/2TRP that harboured some non-favourable UGT1A genotype were more likely to develop severe toxicity at the end of the first cycle of chemotherapy and at the end of treatment (46 *vs* 6% *χ*^2^ *P*=0.018 and 64 *vs* 28% *χ*^2^ *P*=0.12). We did not observe any association between TYMS genotypes and toxicity (data not shown).

### Survival

**Figure 3:**
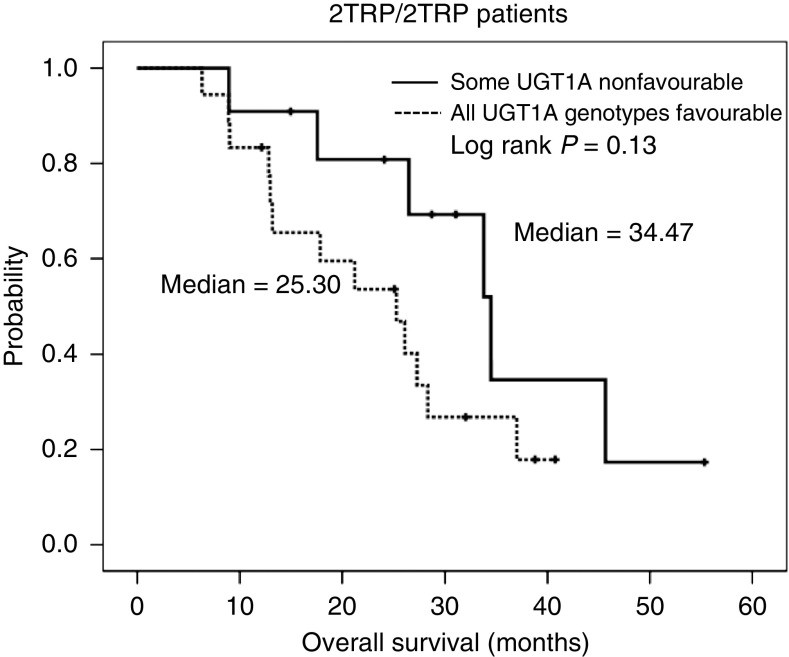
Overall survival curves of patients with some non-favourable (UGT1A1^*^28/^*^28 or UGT1A7^*^3/^*^3 or UGT1A9^*^1/^*^1) UGT1A genotype (solid line) or with all favourable UGT1A genotypes (dotted line). Reported *P*-value corresponds to the log-rank test.

Median TTP and OS were 9.2 months (95% CI=8.4–10.0) and 24.3 months (95% CI=21.8–26.8), respectively. There was no statistical association between any of the genotypes, UGT1A haplotypes or clinical variables and TTP or OS (data not shown). However, patients homozygous for the 2TRP allele had a median OS of 27.3 months (95% CI=23.9–30.7), whereas patients homozygous for the 3TRP allele had a median OS of 21.4 months (95% CI: 19.1–23.7) (HR=1.59; 95% CI: 0.91–2.76; log-rank *P*=0.14). Heterozygous patients had a median OS similar to that of the whole group (24.3 months; 95% CI=19.5–29.2). Interestingly, those patients within the group 2TRP/2TRP with some non-favourable UGT1A genotype had longer OS times (34.5 *vs* 25.3 months, log-rank *P*=0.13) (Figure 3).

## Discussion

The usefulness of genetic testing to predict irinotecan-related toxicities has been widely discussed, but still not clearly convincing as indicated from recent works by Cecchin *et al* (2009) and Braun *et al* (2009). This study helps in identifying those CRC patients with a high risk of developing life-threatening toxicities (i.e., severe neutropenia and diarrhoea) when treated with irinotecan plus 5FU regimens. Our data confirm the role of UGT1A1^*^28 variant as the most important in taking a decision and, for the first time, reveals TYMS 5′TRP polymorphism to be strongly related with tumour response in these patients. Furthermore, this is the third of the largest studies evaluating different UGT1A polymorphisms in Caucasians (Cote *et al*, 2007; Cecchin *et al*, 2009). Owing to the retrospective nature of the study, pharmacokinetic analysis could not be performed, being this fact a weak point of this study.

Among all UGT1A variants studied, UGT1A1^*^28 was the most widely correlated with severe toxicity in the multivariate analysis (see Table 4). It is noteworthy that 33.3% of patients homozygous for the UGT1A1^*^28 allele developed severe neutropenia and diarrhoea at the same time, whereas only 1.8% of UGT1A1^*^1/^*^1 and 5.2% of UGT1A1^*^1/^*^28 patients did so (OR=12.38, 95% CI=2.38–54.18, *P*<0.0001, Supplementary Table S1). Taking into account the high risk of dying of these patients, it has to be considered either not to treat them with irinotecan-containing regimens or to give them a reduced starting dose of irinotecan. UGT1A1^*^28 was specifically associated with neutropenia at the end of treatment and with diarrhoea after first cycle of chemotherapy. Patients homozygous for the UGT1A1^*^28 allele were at a higher risk of developing any kind of severe toxicity at the end of treatment and severe non-haematologic toxicity (specifically diarrhoea) at the end of the first cycle of chemotherapy (Table 5). Our results are somehow in agreement with the majority of the published studies (Marcuello *et al*, 2004; Cote *et al*, 2007; Ruzzo *et al*, 2008; Cecchin *et al*, 2009).

We chose to study UGT1A1^*^28, UGT1A7^*^3 and UGT1A9^*^22 variants based on their functional significance (Iyer *et al*, 1998, 1999; Guillemette *et al*, 2000; Gagne *et al*, 2002; Lankisch *et al*, 2005). It has been suggested that the UGT1A1^*^93 variant might be a better predictor of the UGT1A1 status than the UGT1A1^*^28 (Innocenti *et al*, 2004), but based on two recent studies from the same group (Cecchin *et al*, 2009; Innocenti *et al*, 2009) this possibility remains to be proven. Also it is worth highlighting that the functional significance of this variant is still unknown.

It has been suggested that the effect of UGT1A1^*^28 on haematologic irinotecan-related toxicity is weaker when the drug is administered at intermediate doses (<250 mg m^−2^) (Hoskins *et al*, 2007). Our results do not support this observation given that patients in our study received either 80 mg m^−2^ weekly or 180 mg m^−2^ biweekly of irinotecan and the association with toxicity was strong. Thus, our data should be taken into account together with those from other authors (Marcuello *et al*, 2004; Rouits *et al*, 2004).

UGT1A7^*^3 and UGT1A9^*^22 variants had a minor impact on toxicity and cannot be excluded that the apparent effect could be due to LD with UGT1A1^*^28. Nevertheless, in the multivariate analysis, patients with UGT1A7^*^3/^*^3 and UGT1A9^*^1/^*^1 genotypes were more likely to develop diarrhoea after first cycle of chemotherapy (Table 4). Our results are in agreement with those of Han *et al* (2006). These authors reported an association between these variants and lower area under the time-concentration curve SN-38G to SN-38 ratio. UGT1A9^*^1/^*^1 showed a trend for high incidence of severe diarrhoea (Han *et al*, 2006). In another study, these variants were associated with haematologic toxicity (Cecchin *et al*, 2009).

We hypothesised that harbouring one of these variants would increase the risk of developing treatment-related toxicities. Our hypothesis was confirmed by haplotype and combined genotype analysis. Thus, 7% of patients had all unfavourable genotypes and they experienced higher rates of all severe toxicities at the end of treatment and non-haematologic (specifically diarrhoea) toxicity at the end of the first cycle of chemotherapy (Table 5). Haplotype VII that contains all toxicity-related alleles (UGT1A1^*^28, UGT1A7^*^3 and UGT1A9^*^1) was associated with non-haematologic toxicity both at the end of first cycle and at the end of treatment (Table 5).

Unlike Han *et al* (2006) and Cecchin *et al* (2009) but in agreement with Liu *et al* (2008), we did not find an association of UGT1A genetic variants with either response, TTP or OS.

Interestingly, a strong correlation between TYMS 5′TRP genotypes and response to treatment was observed in our study, both in univariate and multivariate analyses. In a previous study from our group, TYMS genotypes were associated with TTP only in patients treated with 5FU plus oxaliplatin but not in those treated with capecitabine plus oxaliplatin (Martinez-Balibrea *et al*, 2008). Carlini *et al* (2005) also evaluated the relationship between TYMS polymorphisms and response to capecitabine plus irinotecan in metastatic CRC patients, but no significant association was noted. On the basis of these data, we proposed that TYMS genotype might be useful to predict response to 5FU-based schedules.

**Figure 4:**
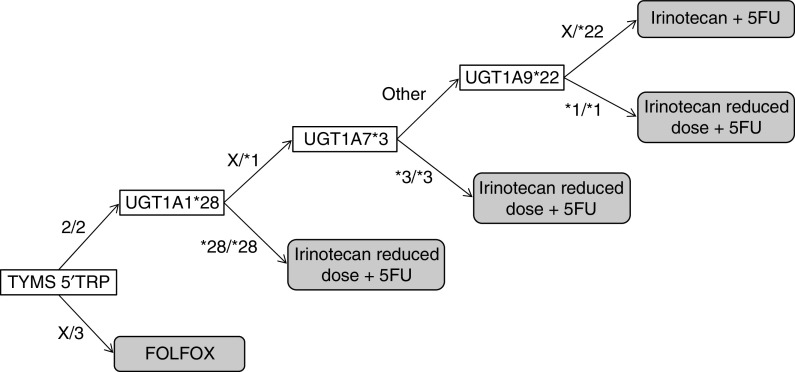
Genetic-based algorithm for first-line treatment selection in colorectal cancer patients. An X in a genotype represents any of the possible alleles. A given patient would be first genotyped for TYMS. If he were heterozygous or homozygous for the 3TRP allele, he would receive an oxaliplatin-based regimen. If he was not, he would be then genotyped for UGT1A1^*^28. If genotype was UGT1A1^*^28/^*^28, this patient would receive a low dose of irinotecan, otherwise he would be genotyped for UGT1A7^*^3. If he were homozygous for the UGT1A7^*^3 allele, he would receive a low dose of irinotecan. Genotypes different from UGT1A7^*^3/^*^3 would be then analysed for UGT1A9^*^22. If the resulting genotype was UGT1A9^*^1/^*^1, this patient would receive a low dose of irinotecan, otherwise he would receive a standard irinotecan plus 5FU schedule.

Taking into account these results, we propose a possible genetic algorithm to select first-line treatment in metastatic CRC patients (Figure 4). First, due to a lack of response, those patients with TYMS genotypes 2TRP/3TRP or 3TRP/3TRP should not receive irinotecan+5FU; second, patients within TYMS 2TRP/2TRP with genotype UGT1A1^*^28/^*^28 or UGT1A7^*^3/^*^3 or UGT1A9^*^1/^*^1 should receive an adjusted dose of irinotecan and be under a stricter surveillance, as they are prone to a higher risk of severe toxicity. In contrast, these patients are more likely to respond and survive longer.

This study is limited by its retrospective design and requires further confirmation in independent studies. If so, our findings might be of clinical value and would help clinicians to better select first-line treatment in advanced CRC patients.

## Figures and Tables

**Table 1:** (I) Genotype and allele frequencies for all polymorphisms, (II) UGT1A haplotypes

**(I) Allele and genotype frequencies**							
**Polymorphism**	**Allele**	***N***	**Freq**	**Genotype**	***N***	**Freq**	**HW**
TS 5′TRP	3TRP	166	0.56	2TRP/2TRP	29	0.2	
	2TRP	130	0.44	2TRP/3TRP	72	0.49	0.87
				3TRP/3TRP	47	0.32	
TS 3′UTR	Ins	207	0.69	Ins/ins	70	0.47	
	Del	91	0.31	Ins/del	67	0.45	0.56
				Del/del	12	0.08	
UGT1A1^*^28	^*^1	190	0.64	^*^1/^*^1	56	0.38	
	^*^28	108	0.36	^*^1/^*^28	78	0.52	0.15
				^*^28/^*^28	15	0.1	
UGT1A9^*^22	^*^1	178	0.60	^*^1/^*^1	57	0.38	
	^*^22	120	0.4	^*^1/^*^22	64	0.43	0.49
				^*^22/^*^22	28	0.19	
UGT1A7^*^3	^*^1	38.5	0.26	^*^1/^*^1	25	0.17	
	^*^2	71	0.48	^*^1/^*^2	20	0.13	
	^*^3	39.5	0.27	^*^1/^*^3	7	0.05	
	^*^4	0	0	^*^2/^*^2	47	0.32	
				^*^2/^*^3	28	0.19	
				^*^3/^*^3	22	0.15	
							
**(II) Haplotype frequencies**							
**Haplotype**	UGT1A1*28	UGT1A9*22	**UGT1A7 N129K/R131K**		**UGT1A7 W208R**	**Freq**	
I	6TA	10dT	TCG		T	0.36	
II	6TA	9dT	GAA		C	0.09	
III	6TA	9dT	GAA		T	0.19	
IV	7TA	10dT	GAA		C	0.01	
V	7TA	10dT	TCG		C	0.00	
VI	7TA	10dT	TCG		T	0.04	
VII	7TA	9dT	GAA		C	0.30	
VIII	7TA	9dT	GAA		T	0.02	

Abbreviations: Freq=frequencies; HW=Hardy–Weinberg equilibrium *P*-value.

UGT1A1^*^1=6TA; UGT1A1^*^28=7TA; UGT1A9^*^1=9dT; UGT1A9^*^22=10dT; See Figure 1 for UGT1A7 alleles definition.

**Table 2:** Clinical features, response and toxicity

	**Response**			**Haematologic toxicity**			**Non-haematologic toxicity**		
***N*=149**	***N* (%)**	**OR (95% CI)**	P*	***N* (%)**	**OR (95% CI)**	P*	***N* (%)**	**OR (95% CI)**	P*
*Gender*									
Male	53 (58.9)	1	0.8	19 (21.1)	1	0.85	35 (38.9)	1	0.6
Female	32 (57.1)	1.07 (0.6–2.1)		13 (22.4)	1.08 (0.49–2.4)		20 (34.5)	0.83 (0.42–1.65)	
									
*Primary tumour*									
Colon	26 (49.1)	1	0.076	9 (17)	1	0.4	22 (41.5)	1	0.52
Rectum	59 (64.1)	0.54 (0.27–1.07)		22 (23.4)	1.5 (0.63–0.35)		34 (36.2)	0.8 (0.4–1.6)	
									
*ECOG*									
0	50 (58.8)	1	0.7	20 (23.4)	1	0.81	36 (42.4)	1	0.28
1	32 (57.1)	1.07 (0.54–2.1)		11 (23.5)	0.76 (0.33–1.7)		18 (31)	0.61 (0.3–1.24)	
2	2 (40)	2.1 (0.3–13.5)		1 (19)	0.81 (0.09–7.6)		1 (20)	0.34 (0.036–3.2)	
									
*Number of cycles*									
⩽3	16 (43.2)	1	0.038	7 (17.9)	1	0.53	14 (35.9)	1	0.8
>3	69 (62.7)	0.45 (0.2–0.97)		25 (22.7)	1.35 (0.65–0.35)		42 (38.2)	1 (0.52–2.4)	
									
*Number of lesions*									
⩽3	45 (54.2)	1	0.31	20 (23.5)	1	0.48	36 (42.4)	1	0.17
>3	40 (62.5)	0.71 (0.37–1.4)		12 (18.8)	0.75 (0.34–1.7)		20 (31.2)	0.62 (0.3–1.22)	
									
*Tumour differentiation*									
Well	29 (69)	1	0.023	11 (26.2)	1	0.4	16 (38)	1	0.01
Moderate	46 (57)	1.7 (0.77–3.7)		17 (21)	0.75 (0.3–1.8)		37 (45.7)	1.4 (0.64–2.9)	
Poor	3 (25)	6.7 (1.6–28.8)		1 (18.3)	0.26 (0.03–2.2)		0	0.62 (0.49–0.79)	
									
*Age (years)*									
⩽65	44 (33)	1	0.2	16 (19)	1	0.4	22 (26)	1	0.001
>65	41 (64)	0.63 (0.32–1.24)		16 (24)	1.4 (0.63–3.04)		34 (52)	3.09 (1.55–6.1)	

Abbreviations: CI=confidence interval; OR=odds ratio.

^*^P-values correspond to *χ*^2^-test or Fisher's exact test, when appropriate.

**Table 3:** Genotypes, haplotypes and response to treatment

**Polymorphism**	**Genotype**	**R (%)**	**NR (%)**	**OR (95% CI)**	***P* ^†^**
	3TRP/3TRP	19 (40)	28 (60)	1.00	
TS 5′TRP	2TRP/3TRP	43 (61)	27 (39)	0.43 (0.20–0.91)	0.0024
	2TRP/2TRP	23 (79)	6 (21)	0.18 (0.06–0.52)	
	Ins/ins	43 (62)	26 (38)	1.00	
TS 3′UTR	Ins/del	36 (55)	30 (45)	1.38 (0.69–2.74)	0.56
	Del/del	6 (50)	6 (50)	1.65 (0.48–5.67)	
	^*^1/^*^1	31 (55)	25 (45)	1.00	
UGT1A1^*^28	^*^1/^*^28	47 (61)	30 (39)	0.79 (0.39–1.59)	0.67
	^*^28/^*^28	7 (50)	7 (50)	1.24 (0.38–4.01)	
	^*^1/^*^1	33 (60)	22 (40)	1.00	
UGT1A9^*^22	^*^1/^*^22	34 (53)	30 (47)	1.32 (0.64–2.74)	0.56
	^*^22/^*^22	18 (64)	10 (36)	0.83 (0.32–2.14)	
	^*^1/^*^1	16 (64)	9 (36)	1.00	
	^*^1/^*^2	10 (50)	10 (50)	1.8 (0.5–5.9)	
UGT1A7	^*^1/^*^3	5 (71.4)	2 (28.6)	0.7 (0.1–4.4)	0.9
	^*^2/^*^2	26 (55.3)	21 (44.7)	1.4 (0.5–3.9)	
	^*^2/^*^3	16 (57.1)	12 (42.9)	1.3 (0.4–4)	
	^*^3/^*^3	12 (60)	8 (40)	1.2 (0.4–4)	
					
**UGT1A Haplotypes^a^**		**OR (95% CI)**			***P***
I		1			
II		1.65 (0.69–3.95)			0.17
III		0.73 (0.37–1.46)			
VII		0.88 (0.48–1.64)			

Abbreviations: CI=confidence interval; NR=no response; OR=odds ratio; R=response.

a Only haplotypes with a frequency >0.05 were considered. ^†^*P*-values correspond to *χ*^2^-test or Fisher's exact test, when appropriate.

**Table 4:** Logistic regression analysis

**Outcome**	**OR (95% CI)**	***P***
*Response rate*		
TS 3TRP/3TRP (ref. 2TRP/2TRP)	5.87 (1.68–20.45)	0.005
Poor differentiation (ref. well)	8.84 (1.79–43.56)	0.007
Number of cycles ⩽3	2.44 (1.02–5.88)	0.046
		
*Severe haematologic toxicity*		
UGT1A1^*^28/^*^28 (ref. ^*^1/^*^28 and ^*^1/^*^1)	6.27 (1.09–36.12)	0.040
		
*Severe non-hematologic toxicity*		
UGT1A9^*^1/^*^1 (ref. ^*^1/^*^22 and ^*^22/^*^22)	2.70 (1.07–6.82)	0.035
Older than 65	3.23 (1.44–7.14)	0.004
		
*Neutropenia*		
UGT1A1^*^28/^*^28 (ref. ^*^1/^*^28 and ^*^1/^*^1)	6.40 (1.11–37.03)	0.038
		
*Neutropenia and diarrhoea*		
UGT1A1^*^28/^*^28 (ref. ^*^1/^*^28 and ^*^1/^*^1)	18.87 (2.14–166.67)	0.008
		
*Diarrhea first cycle*		
UGT1A1^*^28/^*^28 (ref. ^*^1/^*^28 and ^*^1/^*^1)	23.80 (2.05–250)	0.011
UGT1A9^*^1/^*^1 (ref. ^*^1/^*^22 and ^*^22/^*^22)	3.24 (0.99–10.63)	0.053
UGT1A7^*^3/^*^3 (ref. other)	27.64 (1.58–482.86)	0.023

Abbreviations: CI=confidence interval; OR=odds ratio; ref=reference category/categories.

**Table 5:** UGT1A genotypes, haplotypes and toxicity

		**Haematologic**			**No haematologic**			**Neutropenia**			**Diarrhoea**			**Neutropenia and diarrhoea**		
**Gene**	**Genotype**	**Yes (%)**	**OR (95% CI)**	***P***	**Yes (%)**	**OR (95% CI)**	***P***	**Yes (%)**	**OR (95% CI)**	***P***	**Yes (%)**	**OR (95% CI)**	***P***	**Yes (%)**	**OR (95% CI)**	***P***
*First cycle*																
UGT1A1	^*^28/^*^28	2 (13.3)	2.14 (0.42–10.86)	0.31	6 (40)	4.92 (1.55–15.64)	0.004	1 (6.7)	1.13 (0.13–9.67)	1	4 (26.7)	3.7 (1.02–13.42)	0.036	—	—	—
	Other	9 (6.7)	1		16 (11.9)	1		8 (6)	1		12 (9)	1		—	—	
UGT1A9	^*^1/^*^1	3 (5.3)	1	0.44	12 (11.9)	1	0.09	2 (3.5)	1	0.5	9 (15.8)	1	0.1	—	—	—
	Other	8 (8.7)	1.71 (0.44–6.75)		10 (21.1)	0.46 (0.18–1.14)		7 (7.6)	2.3 (0.45–11.3)		7 (7.6)	0.44 (0.15–1.25)		—	—	
UGT1A7	^*^3/^*^3	2 (9.1)	1.31 (0.26–6.52)	0.74	4 (10.9)	1.35 (0.41–4.44)	0.63	8 (4.5)	0.71 (0.08–5–96)	1	2 (9.1)	0.81 (0.17–3.83)	1	—	—	—
	Other	9 (7.1)	1		18 (18.2)	1		1 (6.3)	1		14 (11)	1		—	—	
All UGTs	No favourable^*^	2 (18.2)	3.19 (0.6–17)	0.16	4 (36.4)	3.8 (1.01–14.33)	0.036	1 (9.1)	1.63 (0.18–14.32)	0.5	2 (18.2)	1.97 (0.39–10.03)	0.33	—	—	—
	Other	9 (6.5)	1		18 (13)	1		8 (5.8)	1		14 (10.1)	1		—	—	
All UGTs	Favourable	8 (8.9)	1	0.53	9 (10)	1	0.043	7 (7.8)	1	0.32	6 (6.7)	1	0.047	—	—	—
	Other	3 (5.1)	0.55 (0.14–2.16)		13 (22)	2.54 (1.01–6.41)		2 (3.4)	0.42 (0.08–2.08)		10 (16.9)	2.86 (0.98–8.34)		—	—	
																
*Haplotypes*																
I		—	1	—	—	1	—	—	1	—	—	1	—	—	—	—
II		—	NA	NA	—	0.33 (0.04–2.61)	0.29	—	—	—	—	0.46 (0.06–3.72)	0.47	—	—	—
III		—	0.39 (0.08–1.81)	0.23	—	1.54 (0.61–3.88)	0.36	—	0.45 (0.10–2.06)	0.30	—	1.57 (0.56–4.38)	0.39	—	—	—
VII		—	1.27 (0.48–3.38)	0.63	—	2.5 (1.08–5.81)	0.03	—	0.93 (0.32–2.72)	0.89	—	1.98 (0.76–5.13)	0.60	—	—	—
																
*End of treatment*																
UGT1A1	^*^28/^*^28	6 (40)	2.77 (0.91–8.47)	0.065	10 (66.7)	3.83 (1.23–11–86)	0.014	6 (40)	2.91 (0.95–8.92)	0.05	9 (60)	4.08 (1.36–12.28)	0.008	5 (33.3)	12.7 (3.14–51.34)	<0.0001
	Other	26 (19.4)	1		46 (34.3)	1		25 (18.7)	1		36 (26.9)	1		5 (3.8)	1	
UGT1A9	^*^1/^*^1	10 (17.5)	1	0.36	29 (50.9)	1	0.008	10 (17.5)	1	0.44	24 (42.1)	1	0.013	5 (8.9)	1	0.4
	Other	22 (23.9)	1.48 (0.64–3.4)		27 (29.3)	0.4 (0.2–0.8)		21 (22.8)	1.39 (0.6–3.2)		21 (22.8)	0.41 (0.2–0.8)		5 (5.5)	0.6 (0.16–2.15)	
UGT1A7	^*^3/^*^3	5 (22.7)	1.09 (0.37–3.22)	0.88	12 (54.5)	2.26 (0.91–5.65)	0.075	5 (22.7)	1.14 (0.39–3.39)	0.81	11 (50)	2.74 (1.09–6.89)	0.028	4 (18.2)	4.41 (1.13–17.15)	0.022
	Other	27 (21.3)	1		44 (34.6)	1		26 (20.5)	1		34 (26.8)	1		6 (4.8)	1	
All UGTs	No favourable^*^	5 (45.5)	3.4 (0.97–12.1)	0.044	8 (72.7)	5 (1.27–19.72)	0.02	5 (45.5)	3.59 (1.02–12.67)	0.036	7 (63.6)	4.61 (1.28–16–63)	0.018	4 (36.4)	12.38 (2.83–54.18)	<0.0001
	Favourable	27 (19.6)	1		48 (34.8)	1		26 (18.8)	1		38 (27.5)	1		6 (4.4)	1	
All UGTs	Favourable	21 (21.3)	1	0.5	26 (28.9)	1	0.007	20 (22.2)	1	0.6	20 (22.2)	1	0.009	4 (4.5)	1	0.19
	Other	11 (18.6)	0.75 (0.33–1.71)		30 (50.8)	2.55 (1.29–5.05)		11 (18.6)	0.8 (0.35–1.83)		25 (42.4)	2.57 (1.26–5.27)		6 (10.3)	2.45 (0.66–9.1)	
																
*Haplotypes*																
I		—	1	—	—	1	—	—	1	—	—	1	—	—	1	
II		—	0.16 (0.02–1.24)	0.08	—	1.29 (0.53–3.16)	0.58	—	0.16 (0.02–1.24)	0.08	—	1.27 80.48–3.32)	0.63	—		
III		—	0.58 (0.23–1.43)	0.24	—	1.58 (0.81–3.11)	0.18	—	0.55 (0.22–1.36)	0.2	—	1.31 (0.63–2.71)	0.47	—		
VII		—	1.50 (0.76–2.98)	0.25	—	2.11 (1.12–3.98)	0.02	—	1.52 (0.76–3.03)	0.23	—	2.24 (0.63–7.24)	0.02	—		

Abbreviations: CI=confidence interval; NA=not applicable; OR=odds ratio;

Note: All reported *P*-values correspond to *χ*^2^-test or Fisher's exact test, when appropriate.

No favourable UGT1A genotypes=UGT1A1^*^28/^*^28, UGT1A7^*^3/^*^3 and UGT1A9^*^1/^*^1.
