# Supplementary material for: UGT1A and TYMS genetic variants predict toxicity and response of colorectal cancer patients treated with first-line irinotecan and fluorouracil combination therapy
Source: Br J Cancer. 2010 Jul 13;103(4):581–9. doi: 10.1038/sj.bjc.6605776 (PMC2939780; doi:10.1038/sj.bjc.6605776)
Supplement: Supplementary Table S1 [file 6605776x1.doc]

Supplemental material. Table S1.

Note: all reported p values correspond to chi-square or Fisher’s when appropriate. OR = Odds ratio; CI = Confidence interval.
